# Development of an efficient regeneration and transformation method for the new potential oilseed crop *Lepidium campestre*

**DOI:** 10.1186/1471-2229-13-115

**Published:** 2013-08-12

**Authors:** Emelie Ivarson, Annelie Ahlman, Xueyuan Li, Li-Hua Zhu

**Affiliations:** 1 Department of Plant Breeding, Swedish University of Agricultural Sciences, Box 101, SE 230 53, Alnarp, Sweden

**Keywords:** *Lepidium campestre*, Regeneration, Transformation, Hypocotyls, 2,4-D, TDZ

## Abstract

**Background:**

*Lepidium campestre* is an undomesticated oilseed species with a great potential to become a new crop for both food and industrial feedstocks production. Genetic modification is needed for further improving the oil quantity and quality of Lepidium. Studies on in vitro shoot regeneration of Lepidium are very limited and there is no transformation protocol available.

**Results:**

We have investigated the effects of different factors, especially the type, concentration and combination of plant growth regulators (PGRs) on in vitro shoot regeneration of Lepidium. The results showed that the 2,4-D treatment was crucial to shoot regeneration from different explants. The duration of 2,4-D exposure between 2-4 days did not show significant difference in shoot regeneration, while the effect of 2,4-D concentration varied greatly depending on the type of explants and cytokinins used, for example, the low concentration of 2,4-D combined with TDZ significantly increased the regeneration frequency of hypocotyls. Cotyledon and hypocotyl explants responded differently to cytokinin, for example, TDZ was more effective than zeatin in promoting shoot regeneration from hypocotyls, but did not affect the regeneration of cotyledons which was more affected by high concentration of zeatin. The results also showed that NAA was not effective for shoot regeneration. Germination in light increased the regeneration frequency compared to that in dark. After optimization of the different conditions, an efficient regeneration protocol was developed with the regeneration efficiency of 92.7%. Using this protocol, the transformation frequency of 6% in average was achieved. The presence of transgenes in the transgenic lines was confirmed by GUS staining, PCR and Southern blot analyses.

**Conclusion:**

Through systematic investigation of important factors affecting in vitro shoot regeneration, we have developed an efficient regeneration and transformation protocol for the genetic modification of *Lepidium campestre.* The method may also be applied to the related species.

## Background

As fossil oil reserves decline and the climate changes, the need for environmental friendly and renewable plant oils for both food and industry purposes has become ever more imperative [1-3]. Improving the current oil crops and developing new and more productive oilseed crops with multiple functions have thus become more attractive as the potential of increasing production of the existing oilseed crops is limited.

*Lepidium campestre*, belonging to the Brassicaceae family, has a great potential to become a new crop for plant oil production. Lepidium has a high yield potential (5 ton/ha, ca. 30% higher than the average of winter oilseed rape). It is very cold-hardy and could be grown in the regions where winter oilseed rape cannot be cultivated, thus greatly expanding the planting regions of oil crops. It has good agronomic traits, such as, an upright stature, branching only in the upper part of the stem and resistance to the pollen beetle [4]. Duo to its biennial nature, Lepidium is also a good catch crop which has shown a positive effect on the seed yield of the mother crop barley [4,5]. This cropping system could reduce the use of tillage and thus reducing energy-consumption. The seed oil of Lepidium contains some special components, for example, high content of erucic acid, an important industrial feedstock [6,7]. Moreover, self-fertilisation and diploidy of Lepidium are also important traits that could facilitate its genetic modification.

Lepidium is an undomesticated oilseed species. It has some problems that need to be solved before any commercialization, for instance, it contains low oil content ca. 20% compared to ca. 45% in rapeseed. Moreover, the seed oil composition of Lepidium needs further improvement in order to meet the special requirements for different industries.

Genetic engineering offers a more precise and efficient approach for improving important agronomic traits, especially for qualitative traits. However, an efficient regeneration protocol is the prerequisite for a successful genetic modification. For Lepidium*,* studies on in vitro regeneration and transformation are very limited. It has been previously reported that a regeneration frequency of 68.3% is achieved when using cotyledons as explants on medium containing zeatin after initial 2,4-D induction [8]. However, this efficiency is still not high enough for transformation as the regeneration efficiency is normally reduced considerably after *Agrobacterium* infection. Moreover, there is no any transformation protocol available for Lepidium. In this study, we have systemically investigated some important factors affecting the shoot regeneration of Lepidium and successfully developed an efficient regeneration and transformation protocol for this species.

## Methods

### Plant material

Seeds from *L. campestre* accession no. NO94-7 were used and this accession was initially collected in Öland, Sweden and further multiplied in greenhouse.

### Culture conditions

All in vitro cultures from germination to rooting were maintained in a growth chamber with a 16 h day length at 33 μmol m^-2^ s^-1^ and temperature 21/18°C (day/night).

### Germination

Seeds were surface-sterilized with 6% calcium hypochlorite for 20 min, rinsed thoroughly with sterilized water and germinated in vitro on the medium containing the half strength of MS [9] with MES, 1% (w/v) sucrose, pH 5.8 for a couple of days in dark or light depending on experiment (see the result section for details).

### Shoot regeneration

The cotyledons and hypocotyls from 6 days-old seedlings grown in light unless otherwise stated were used as explants in this study. The wounded cotyledons were horizontally placed on the regeneration medium, while the entire hypocotyls were horizontally placed on the regeneration medium after removing of the cotyledons, shoot meristem and root. All media used in this study unless otherwise stated consisted of MS with MES, pH5.8, supplemented with 30 g L^-1^ sucrose and 2.5 g L^-1^ Gelrite and this medium is called the basal MS medium hereafter. The types and concentrations of plant growth regulators (PGRs) used depend on experiment (see the result section for details). The explants were transferred to fresh medium every 3 weeks for regeneration tests and 2 weeks for transformation experiments. The regeneration results were recorded after 3.5 months. For all regeneration tests, the number of explants was at least 30 for each treatment and all tests were repeated at least twice. The regeneration frequency was calculated through dividing the number of regenerated explants by total number of explants used. Normally each explant produces only one rosette which is considered as one shoot.

### Kanamycin resistance test

The hypocotyls from 6 days-old seedlings grown in light were used as explants and pre-cultured for 3 days on the basal MS medium, supplemented with 0.5 mg L^-1^ 2,4-D (2,4-Dichlorophenoxyacetic acid), followed by transfer to the basal MS medium, supplemented with 1.1 mg L^-1^ TDZ (thidiazuron) and kanamycin at the concentrations of 0, 20, 30 and 50 mg L^-1^. For each concentration, about 100 hypocotyls were used and the test was repeated twice. The explants were transferred to fresh medium every 3 weeks and the regeneration results were recorded after 3.5 months.

### Transformation

*Agrobacterium tumefaciens* strain AGL-1 was used for transformation. Since the aim of this study was to work out an efficient transformation protocol, we used the binary vector pSCV1.6 [10] containing only *neomycin phosphotransferase* (*nptII*) and *β-glucuronidase* (*gus*) genes. In this vector, the *gus* gene with an intron is under the 35S promoter and the *nptII* gene under the *nos* promoter.

Hypocotyls from 6 days old seedlings grown in light were used as explants. The explants were pre-cultured for 2 days on a filter paper on the pre-culture medium (the basal MS medium, supplemented with 0.5 g L^-1^ 2,4-D). The reason for using the filter paper was to facilitate the transfer of the explants for the infection. For transformation, the bacteria were cultured in the liquid LB medium with appropriate antibiotics for about 18 h. After centrifuge at 3,500 rpm for 15 min, the pellet was suspended in the liquid MS20 medium (MS with MES, 20 g L^-1^ sucrose, pH 5.2) to a concentration around 0.5 at OD_600_. The pre-cultured explants were thoroughly washed in the bacterial suspension, dry blotted on filter paper and co-cultured on filter paper on the co-culture medium for 4 days in light. The co-culture medium consisted of the basal MS medium, supplemented with 1.1 mg L^-1^ TDZ. After co-culture, the explants were washed with the liquid MS20 medium, dry blotted on filter paper and placed on the selection medium, which was the same as the co-culture medium, but supplemented with 150 mg L^-1^ ticarcillin and 25 mg L^-1^ kanamycin. Alternatively, the explants were selected on the medium containing 15 mg L^-1^ kanamycin for the first subculture, followed by 25 mg L^-1^ kanamycin for the second subculture and 30 mg L^-1^ kanamycin for the subsequent subcultures. The explants were transferred to the fresh selection medium every 2 weeks and cultured in the climate chamber as stated above. The transformation frequency was calculated by dividing the number of explants that produced transgenic shoots by the total explants used for *Agrobacterium* infection. Normally only one shoot was produced per explant.

### Shoot proliferation and rooting

Once the regenerated shoots were about 0.5 cm in size, they were excised from the hypocotyl explants and cultured on the elongation medium as described by Li et al. [11,12]. When shoots reached about 2 cm in height, they were rooted on the rooting medium according to Li et al. [11,12].

### GUS staining

To evaluate the transformation events at early stages, the leaves of putative transgenic shoots were collected for GUS staining according to Jefferson et al. [13].

### PCR and Southern blot analysis

Only the plants which grew well on the selection medium for at least two months were further used for both PCR and Southern blot analysis. The total genomic DNA was extracted from in vitro grown shoots using the CTAB method as described by Aldrich and Cullis [14].

For PCR analysis, both *nptII* and *gus* genes were analyzed to verify integration of the transgenes. The primers used were: for the *nptII* gene, 5′-GCCCTGAATGAACTGCAGGACGAGGC-3′ and 5′-GCAGGCATCGCCATGGGTCACGACGA-3′, yielding a 411 bp product; for the *gus* gene, 5′-CCTGTAGAAACCCCAACCCGTG-3′ and 5′-CCCGGCAATAACATACGGCGTG-3′, yielding a 365 product. The PCR analysis was performed according to Zhu and Welander [15].

Southern blot analysis was performed to confirm the transformation events and to detect the copy number of the transgenes. About 20 μg genomic DNA were digested with the restriction enzyme *Eco*RI that cuts only once on the T-DNA of the pSCV1.6 vector without cutting the *nptII* gene. Southern blot hybridization was based on the non-radioactive DIG system from Roche (Van Miltenburg [16]) and the *nptII* probe was synthesized using the same primer set as for PCR according to Zhu et al. [17].

### Statistic analysis

Data from all regeneration tests were statistically analyzed with ANOVA with Turkey’s procedure using the Statgraphics Plus 5.1 program.

## Results and discussion

### Effects of PGR combinations on shoot regeneration

In vitro organogenesis from mature tissues often requires dedifferentiation of cultured cells. It has been reported that auxin induces the callus formation by initiating the cell dedifferentiation process, while cytokinin stimulates organ regeneration by promoting cell division and differentiation [18]. The ratio of these two PGRs plays an important role in shoot regeneration [19]. As effects of PGRs and their ratios are often species dependent, a number of tests were carried out in this study to optimize the PGR conditions for shoot regeneration in Lepidium.

Previous studies on other plant species have showed that the combinations of cytokinin (mostly BAP (6-benzylaminopurine), TDZ and zeatin) and auxin (mostly 2,4-D, NAA (1-naphthaleneacetic acid) and IAA (indole-3-acetic acid)) normally resulted in reasonable shoot regeneration frequencies [11,15,20-23]. In this study, we initially tested a number of concentrations and combinations of cytokinin and auxin. The results showed that most combinations did not work in Lepidium as no shoot regeneration was observed except for the combination of TDZ 0.44 mg L^-1^ with NAA 0.2 mg L^-l^ where only 10% of regeneration frequency was achieved (Table 1). However, the combinations of 2, 4-D with zeatin or TDZ gave a promising result, for example, the regeneration frequency of the combination of 2, 4-D with TDZ reached 76.5% when using hypocotyls as explants, suggesting that 2, 4-D is an suitable type of auxin, while NAA did not seem to be effective for *in vitro* shoot regeneration of Lepidium. This result is in line with that reported by Eriksson and Merker [8] where 2, 4-D was proved to be essential for shoot regeneration of *L. campestre*.

**Table 1:** Shoot regeneration from cotyledons (C) and hypocotyls (H) of*L. campestre*grown on the media containing different plant growth regulators*

Treatment **	Shoot regeneration %***
	**Means ± SD**
Zeatin 1.0 + NAA 0.5, C	0.0 ± 0.00 e
Zeatin 1.0 + NAA 0.5, H	0.0 ± 0.00 e
Zeatin 2.0 + NAA 0.5, C	0.0 ± 0.00 e
Zeatin 2.0 + NAA 0.5, H	0.0 ± 0.00 e
Zeatin 5.0 + NAA 0.5, C	0.0 ± 0.00 e
Zeatin 5.0 + NAA 0.5, H	0.0 ± 0.00 e
TDZ 0.44 + NAA 0.2, C	0.0 ± 0.00 e
TDZ 0.44 + NAA 0.2, H	10.0 ± 0.30 d
TDZ 1.1 + NAA 0.2, H	0.0 ± 0.00 e
TDZ 2.2 + NAA 0.2, C	0.0 ± 0.00 e
TDZ 2.2 + NAA 0.2, H	0.0 ± 0.00 e
Zeatin 5.0→TDZ 5.0, H	0.0 ± 0.00 e
2,4-D 0.5, 2 days→zeatin 2.0, H	48.3 ± 0.50 b
2,4-D 1.0, 2 days→zeatin 2.0, H	28.6 ± 0.45 c
2,4-D 1.0, 2 days→TDZ 1.1, H	76.5 ± 0.46 a

*Each treatment contained 30 explants and repeated at least twice and the data are means ±SD. **Figure after each PGR indicates the concentration in mg L^-1^. ***Means followed by different letters indicate significant differences at p = 0.05.

### Effects of duration of 2,4-D exposure on shoot regeneration

The effect of 2,4-D on in vitro regeneration has been widely reported in various plant species [24-27]. As longer exposure of 2,4-D could inhibit the shoot formation or even cause mutation, we therefore examined effects of 2,4-D duration on shoot regeneration of Lepidium. The results showed that the durations of 2, 3 and 4 days of 2,4-D exposure did not show significant differences on shoot regeneration frequency for hypocotyls, but for cotyledons, 2,4-D exposure for 1 day resulted in lower regeneration frequency compared with 3 days (Table 2). An interesting finding is that, when using low concentration of zeatin (0.5 mg L^-1^), shoot regeneration could occur from cotyledons although at very low efficiency, while no any regeneration occurred from hypocotyls. This result was further confirmed in the subsequent experiments shown later in this study.

**Table 2:** **Shoot regeneration from cotyledons (C) and hypocotyls (H) of*L. campestre*cultured on the media containing 1.0 mg L**^**-1**^**2,4-D (2,4-D1.0) for 1 (1d), 2 (2d), 3 (3d) and 4 (4d) days before being transferred to 0.5 mg L**^**-1**^**zeatin (z 0.5) for 4 weeks (4w), followed by 2.0 mg L**^**-1**^zeatin (z 2.0)*

**Treatment**	Shoot regeneration %**
	**Means ± SD**
2,4-D 1.0, 1d → z 0.5, 4w→z 2.0, C	6.5 ± 0.25 b
2,4-D 1.0, 1d → z 0.5, 4w→z 2.0, H	0.0 ± 0.00 c
2,4-D 1.0, 2d → z 0.5, 4w→z 2.0, C	9.4 ± 0.30 ab
2,4-D 1.0, 2d → z 0.5, 4w→z 2.0, H	0.0 ± 0.00 c
2,4-D 1.0, 3d → z 0.5, 4w→z 2.0, C	14.0 ± 0.35 a
2,4-D 1.0, 3d → z 0.5, 4w→z 2.0, H	0.0 ± 0.00 c
2,4-D 1.0, 4d → z 0.5, 4w→z 2.0, C	19.4 ±0.40 a
2,4-D 1.0, 4d → z 0.5, 4w→z 2.0, H	0.0 ± 0.00 c

*Each treatment contained 30 explants and repeated at least twice and the data are means ±SD. **Means followed by different letters indicate significant differences at p = 0.05.

### Effects of 2,4-D concentration on shoot regeneration

To evaluate the effect of 2,4-D concentration on shoot regeneration, two concentrations of 2,4-D in the pre-culture medium were tested. The results showed that, when using 1.0 mg L^-1^ 2,4-D, all treatments resulted in poor or no regeneration (with the highest regeneration frequency of 27.5%) regardless type of explants or cytokinin used. However, when the 2,4-D concentration was decreased to 0.5 mg L^-1^, the treatment of 1.1 mg L^-1^ TDZ resulted in 92.7% of regeneration frequency from hypocotyls, the highest among all treatments in this study, while still no generation occurred from cotyledons with TDZ or zeatin treatments and from hypocotyl with zeatin treatment (Table 3). These results indicate that the effect of 2,4-D concentration on shoot regeneration depends not only on type of explants but also on type of cytokinin used. Eriksson and Merker [8] reported the highest regeneration frequency of 68.3% from cotyledons but no regeneration was obtained from hypocotyls with 1.0 mg L^-1^ 2,4-D and 0.5 mg L^-1^ zeatin. The failure of regeneration from hypocotyls in this case might be due to higher level of 2,4-D (1.0 mg L^-1^) in combination with the use of zeatin. It has been suggested that a threshold intracellular concentration of 2,4-D is probably required at each stage of morphogenesis in the tissue culture of different species, for example, callus initiation required a relatively high level of 2,4-D and rhizogenesis occurred only when 2,4-D in the calli declined to a certain level, while shoot buds were initiated at a undetectable concentration of 2,4-D [24,26]. These morphogenic events were achieved through gradual uptake and metabolism of 2,4-D during cell division and callus growth as well as the continuous decline of 2,4-D level due to detoxification or degradation [28-30]. The results of this study have shown that this threshold is also related to the type of explants and cytokinins used. Furthermore, our results suggest that TDZ is a better type of cytokinin for shoot regeneration from hypocotyls, while zeatin might be better for shoot regeneration from cotyledons of Lepidium which is in line with the previous study on cotyledons with zeatin reported by Eriksson and Merker [8].

**Table 3:** **Shoot regeneration from cotyledons (C) and hypocotyls (H) of*L. campestre*cultured on the media containing either 0.5 or 1.0 mg L**^**-1**^**2,4-D (2,4-D 0.5 or 2,4-D1.0) for 3 days (3d), followed by transfer to the medium containing either 1.0 mg L**^**-1**^**zeatin (z1.0), or 1.1 mg L**^**-1**^TDZ (TDZ1.1)*

**Treatment**	Shoot regeneration %**
	**Means ± SD**
2,4-D 0,5, 3d→z 1.0, C	0.0 ± 0.00 d
2,4-D 0,5, 3d→z 1.0, H	0.0 ± 0.00 d
2,4-D 1.0, 3d→z 1.0, C	5.0 ± 0.22 c
2,4-D 1.0, 3d→z 1.0, H	2.7 ± 0.16 c
2,4-D 0,5, 3d→TDZ 1.1, C	0.0 ± 0.00 d
2,4-D 0,5, 3d→TDZ 1.1, H	92.7 ± 0.26 a
2,4-D 1.0, 3d→TDZ 1.1, C	0.0 ± 0.00 d
2,4-D 1.0, 3d→TDZ 1.1, H	27.5 ± 0.45 b

*Each treatment contained 30 explants and repeated at least twice and the data are means ±SD. **Means followed by different letters indicate significant differences at p = 0.05.

### Effects of TDZ on shoot regeneration

The effect of TDZ on shoot regeneration is well documented [31]. Our results in this study have shown that the combinations of 2,4-D and TDZ resulted in better shoot regeneration frequencies compared with those of 2,4-D and zeatin. To determine the suitable level of TDZ for Lepidium regeneration, different concentrations of TDZ combined with 2,4-D in comparison with zeatin treatments were investigated. The results show that the best combination for shoot regeneration was using hypocotyls as explants with the pre-culture on 0.5 mg L^-1^ 2,4-D for 2 days, followed by transfer to 1.1 mg L^-1^ TDZ (Table 4). This combination was thus chosen for the subsequent transformation experiments. It should be noticed that in this study auxin was not added in the medium after the removal of 2,4-D, but auxin is normally required for organogenesis or somatic embryogenesis. The explanation of this might be either due to the post-effect of 2,4-D or because TDZ has a dual role in the organogenesis, namely, both promoting the cell division and differentiation as well as inducing dedifferentiation [32]. Many studies have shown that TDZ is involved in the modulation of endogenous phytohormones, especially auxins and cytokinins [33-37].

**Table 4:** **Shoot regeneration from cotyledons (C) and hypocotyls (H) of*L. campestre*cultured on the medium containing 0.5 mg L**^**-1**^**2,4-D (2,4-D 0,5) for 1, 2 or 3 days (1d, 2d or 3d), followed by transfer to the media containing either 1.0 mg L**^**-1**^**zeatin (z1.0) or 1.1 mg L**^**-1**^TDZ (TDZ1.1)*

**Treatment**	Shoot regeneration %**
	**Means ± SD**
2,4-D 0,5, 1d→Z 1.0, C	3,3 ± 0.18 d
2,4-D 0,5, 1d→Z 1.0, H	52.1 ± 0.50 c
2,4-D 0,5, 2d→Z 1.0, C	1.6 ± 0.13 d
2,4-D 0,5, 2d→Z 1.0, H	65.8 ± 0.48 bc
2,4-D 0,5, 1d→TDZ 1.1, C	1.7 ± 0.13 d
2,4-D 0,5, 1d→TDZ 1.1, H	88.0 ± 0.33 a
2,4-D 0,5, 2d→TDZ 1.1, C	3.2 ± 0.18 d
2,4-D 0,5, 2d→TDZ 1.1, H	83.6 ± 0.37 ab
2,4-D 0,5, 3d→TDZ 1.1, C	0.0 ± 0.00 d
2,4-D 0,5, 3d→TDZ 1.1, H	92.7 ± 0.26 a

*Each treatment contained 30 explants and repeated at least twice and the data are means ±SD. **Means followed by different letters indicate significant differences at p = 0.05.

### Effects of stepwise increase in cytokinin concentration on shoot regeneration

It is well-known that high auxin concentrations stimulate callus formation, but inhibit shoot regeneration. In most studies a high auxin/cytokinin ratio is usually used for inducing callus formation. Thereafter the auxin level must be reduced while increasing the cytokinin level for stimulating shoot formation. Based on this theory, a stepwise increase in cytokinin concentration after pre-culture with 2,4-D was studied to evaluate the effect of such a treatment on shoot regeneration. Considering that TDZ has a dual role in organogenesis, only zeatin in combination with different durations of pre-culture with 2,4-D was tested. The regeneration frequencies (67.0% and 29.8%) were obtained when culturing hypocotyls on the pre-culture medium with 0.5 or 1.0 mg L^-1^ 2,4-D for 2 days, followed by a transfer to 1.0 mg L^-1^ zeatin for 6 weeks before being transferred to 2.0 mg L^-1^ zeatin, while low or very low regeneration frequencies were obtained with the treatments where 1.0 mg L^-1^ zeatin was maintained only for 2 weeks before being transferred to 2.0 mg L^-1^ zeatin (Table 5). These results indicate that, after the 2,4-D treatment, a relatively longer time with low level of cytokinin (1.0 mg L^-1^) was probably needed for the initiation of cell dedifferentiation and callus formation, and thereafter a high concentration of cytokinin (2.0 mg L^-1^) for organ differentiation and shoot formation from hypocotyl explants.

**Table 5:** **Shoot regeneration from cotyledons (C) and hypocotyls (H) of*L. campestre*cultured on the media containing either 0.5 or 1.0 mg L**^**-1**^**2, 4-D (2, 4-D 0, 5 and 2, 4-D1.0) for 2 or 3 days (2d and 3d), followed by transfer to 1.0 mg L**^**-1**^**zeatin (z1.0) for 2 or 6 weeks (2w and 3w) before being transferred to 2.0 mg L**^**-1**^zeatin (z 2.0)*

**Treatment**	Shoot regeneration %**
	**Means ± SD**
2,4-D 0,5, 3d→z 1.0, 2w→z 2.0, C	4,7 ± 0.21 c
2,4-D 0,5, 3d→z 1.0, 2w→z 2.0, H	7.2 ± 0.26 c
2,4-D 0,5, 2d →z 1.0, 6w→z 2.0, C	1.0 ± 0.10 d
2,4-D 0,5, 2d →z 1.0, 6w→z 2.0, H	67.0 ± 0.47 a
2,4-D 1.0, 3d→z 1.0, 2w→z 2.0, C	3.3 ± 0.18 c
2,4-D 1.0, 3d→z 1.0, 2w→z 2.0, H	0.0 ± 0.00 d
2,4-D 1.0, 2d →z 1.0, 6w→z 2.0, C	1.0 ± 0.10 d
2,4-D 1.0, 2d →z 1.0, 6w→z 2.0, H	29.8 ± 0.45 b

*Each treatment contained 30 explants and repeated at least twice and the data are means ±SD. **Means followed by different letters indicate significant differences at p = 0.05.

### Effects of dark treatment on shoot regeneration

Dark treatment of mother plants or explants can stimulate shoot regeneration in some species [15,20,38]. In this study, we compared the effects of seed germination in dark and light on shoot regeneration using different plant growth regulator combinations. The results showed that germination in light resulted in better regeneration frequency, for example, the combination of TDZ with the light treatment gave 30% regeneration from hypocotyls, while no or much lower regeneration frequency for the dark treatment (Table 6). Besides, the hypocotyls from the dark-grown seedlings were very weak and difficult to handle. Our results also showed that the low concentration of zeatin favored shoot regeneration from cotyledons, while TDZ promoted shoot regeneration from hypocotyls (Table 6), a similar trend shown in Tables 2 and 3.

**Table 6:** **Shoot regeneration from cotyledons (C) or hypocotyls (H) of*L. campestre*cultured on the medium containing 1.0 mg L**^**-1**^**2,4-D (2,4-D1.0) for 3 days (3d), followed by transfer to 0.5 mg L**^**-1**^**zeatin for 2 weeks (z 0.5, 2w), then to 2.0 mg L**^**-1**^**zeatin (z 2.0) or 0.44 mg L**^**-1**^**TDZ for another 2 weeks (TDZ 0.44, 2w), then to 1.1 mg L**^**-1**^TDZ*

Treatment**	Regeneration %***
	**Means ± SD**
2,4-D1.0, 3d →z 0.5, 2w→z 2.0, C, L	10.4 ± 0.30 b
2,4-D1.0, 3d →z 0.5, 2w→z 2.0, H, L	0.0 ± 0.00 c
2,4-D1.0, 3d →z 0.5, 2w→z 2.0, C, D	6.4 ± 0.25 b
2,4-D1.0, 3d →z 0.5, 2w→z 2.0, H, D	0.0 ± 0.00 c
2,4-D1.0, 3d →TDZ 0.44, 2w→TDZ 1.1, C, L	0.0 ± 0.00 c
2,4-D1.0, 3d →TDZ 0.44, 2w→TDZ 1.1, H, L	30.0 ± 0.46 a
2,4-D1.0, 3d →TDZ 0.44, 2w→TDZ 1.1, C, D	0.0 ± 0.00 c
2,4-D1.0, 3d →TDZ 0,44, 2w→TDZ 1.1, H, D	11.1 ± 0.42 b

*The seeds were germinated either in light (L) or dark (D).

**Each treatment contained 30 explants and repeated at least twice and the data are means ±SD. ***Means followed by different letters indicate significant differences at p = 0.05.

### Kanamycin resistance test

In this study, the kanamycin test showed clearly that the regeneration frequency decreased significantly with an increase in kanamycin concentration between 10-30 mg L^-1^. When the kanamycin concentration reached 30 mg L^-1^, the regeneration frequency was reduced to 13%, while only very limited regeneration was observed when the kanamycin concentration reached 50 mg L^-1^. The cultures became yellowish after 6 weeks when the kanamycin concentration was 10 mg L^-1^, but after 4 weeks for the 20-50 mg L^-1^ treatments (Table 7). We have therefore chosen the kanamycin concentration up to 30 mg L^-1^ in the subsequent transformation experiments.

**Table 7:** **Shoot regeneration from hypocotyls of*L. campestre*cultured on the 0.5 mg L**^**-1**^**2,4-D pre-culture medium for 3 days, followed by transfer to 1.1 mg L**^**-1**^TDZ, supplemented with different concentrations of kanamycin*

**Kanamycin concentration (mg L**^**-1**^**)**	Shoot regeneration % **
	**Mens ± SD**
0	94.0 ± 0.24 a
10	56.0 ± 0.50 b
20	31.0 ± 0.46 c
30	13.0 ± 0.33 d
50	0.9 ± 0.10 d

*Each treatment contained 30 explants and repeated twice and the data are means ±SD. **Means followed by different letters indicate significant differences at p = 0.05.

### Transformation efficiency

Through using the best regeneration protocol achieved from this study, namely, hypocotyls from 6 days old light-grown seedlings pre-cultured on the basal MS medium, supplemented with 0.5 mg L^-1^ 2,4-D for 2 days, followed by transfer to 1.1 mg L^-1^ TDZ, we carried out a number of Lepidium transformations. The kanamycin selection was carried out either at the constant concentration of 25 mg L^-1^ or 15 mg L^-1^ for 2 weeks, followed by a stepwise increase up to 30 mg L^-1^ with each subculture. The transformation efficiency varied among different transformations, ranging from 2.9 to 10.7 with an average about 6% and standard deviation of 2, 87 of 8 independent transformations with about 200 explants each. Compared with our results, the failure in *Agrobacterium* transformation of Lipidium reported by Eriksson and Merker [8] appears to be non-optimized conditions including the type of explants, *Agrobacterium* strain and PGR as well as the concentration of 2,4-D for regeneration and transformation.

### Recovery, shoot proliferation and rooting of transgenic lines

**Figure 1:**
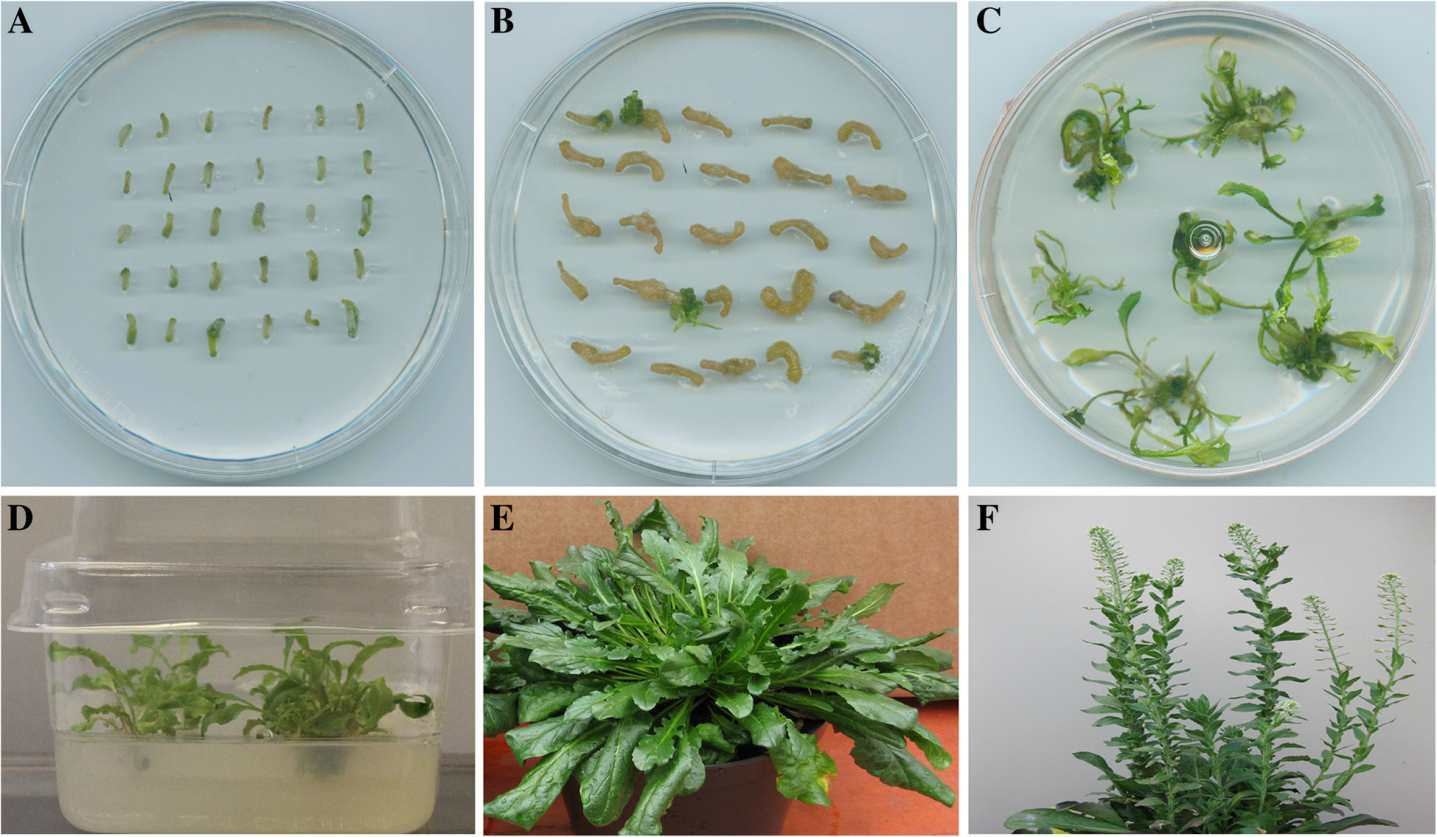
**Different stages of genetic transformation of *L. campestre. *A** = start of hypocotyl pre-culture. **B**= 4 weeks after the *Agrobacterium* infection of hypocotyl explants. **C**= putative transgenic shoots grown on the regeneration medium. **D**= putative shoots grown on the shoot multiplication medium. **E**= a transgenic plant established in greenhouse. **F**= a flowering transgenic plant grown in biotron.

Shoot regeneration started to occur 4 - 6 weeks after *Agrobacterium* infection. However, the most shoot regeneration happened between 8-10 weeks, while this regeneration lasted even after 3-4 months. Transgenic shoots could proliferate well on the shoot production medium. The rooting percentage of transgenic lines on the rooting medium was close to 100% and the establishment of plantlets in greenhouse was 100% (Figure 1). Rooting of the transgenic lines could happen automatically on the proliferation medium in some cases.

### GUS staining

**Figure 2:**
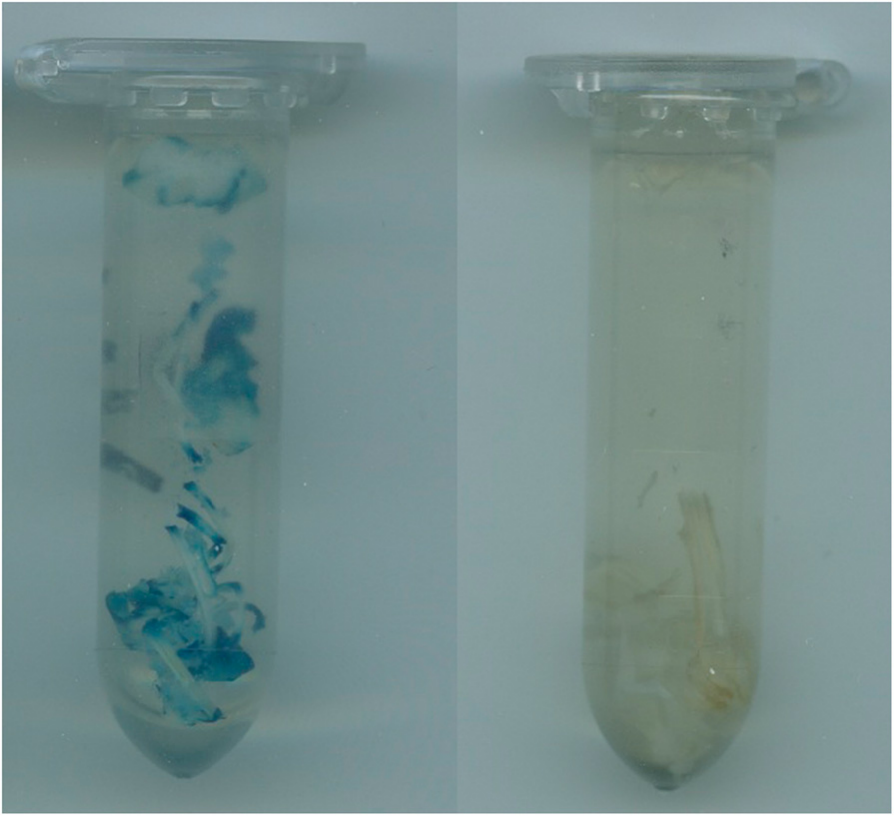
**GUS staining from in vitro shoots of one transgenic line (left) and non-transgenic control (right) of *L. campestre.***

The putative transgenic lines were first verified by GUS staining before sufficient amount of plant materials were obtained for PCR and Southern blot analysis. The strong GUS expression was observed in the leave and shoots of the putative transgenic lines, while no blue color was observed for the non-transgenic control (Figure 2).

### PCR and Southern analyses

**Figure 3:**
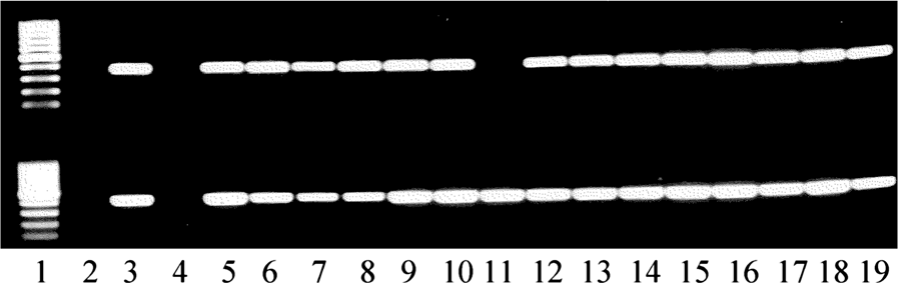
**PCR results of the *gus *(upper panel) and *nptII *(lower panel) genes from the transgenic lines (Lanes 5-19) and non-transgenic control (Lane 4) of *L. campestre.*** Lane 1 = markers, Lane 2 = blank, Lane 3 = plasmid DNA.

**Figure 4:**
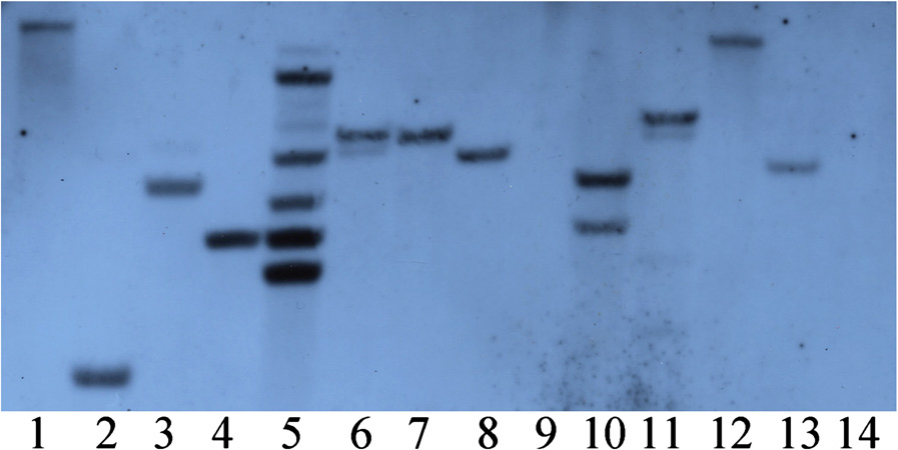
**Southern blot results of transgenic lines (Lanes 1-13) and non-transgenic control (Lane 14) of *L. campestre.*** The DNA was digested with the *Eco*RI and hybridized with the DIG-labeled *nptII* probe.

The PCR results on the genomic DNA of transgenic lines showed that the integration of both *nptII* and *gus* genes (Figure 3). As all tested clones were PCR positive, the plants which grew well on selection medium for more than two months are considered as transgenic lines and thus used for calculating transformation efficiency. Southern blot analysis showed clear bands when hybridized with the *nptII* probe, indicating the stable integration of the T-DNA into the Lepidium genome (Figure 4). The band patterns in Figure 4 showed that the copy number of the *nptII* gene ranged from 1 to at least 5, but most of the transgenic lines tested showed a single copy of the transgene.

## Conclusion

Lepidium is a new promising oilseed species for plant oil production for food and industrial purposes as well as a catch crop. The species has some important agronomic traits, like high seed yield potential, winter hardiness and upright growth habit, but has also some problems that need to be further improved. There is no efficient regeneration and transformation protocol available for this species. Through systematic investigation of important factors affecting in vitro shoot regeneration, we have developed an efficient regeneration and transformation protocol for Lepidium, which would facilitate its improvement of oil quantity and quality through genetic modification in the future.

## Abbreviations

2,4-D: 2,4-dichlorophenoxyacetic acid; GUS: *β*-glucuronidase; NAA: α-naphthaleneacetic acid; NptII: Neymycin phosphotransferase; TDZ: Thidiazuron.

## Competing interests

The authors declare that they have no competing interests.

## Authors’ contribution

EI performed the experimental work, molecular analyses and participated in writing of the manuscript. AA performed the experimental work and some molecular analyses. XL performed the statistical analysis, participated in the design and writing of the manuscript. LZ led the project, designed and coordinated the experimental work, designed the draft of the manuscript, written the final version of the manuscript. All authors read and approved the final manuscript.
